# An evidence-based care program in a local healthcare setting in Brazil: Experience and impact

**DOI:** 10.1016/j.clinsp.2025.100640

**Published:** 2025-04-16

**Authors:** César Ramos Rocha-Filho, Felipe Sebastião de Assis Reis, Aline Rocha, Ana Carolina Pereira Nunes Pinto, Rozana Mesquita Ciconelli

**Affiliations:** a Research and Education Department, BP ‒ A Beneficência Portuguesa de São Paulo, São Paulo, SP, Brazil; b Health Technology Assessment Center, BP ‒ A Beneficência Portuguesa de São Paulo (NATS BP), São Paulo, SP, Brazil; c Department of Medical Practices, BP ‒ A Beneficência Portuguesa de São Paulo, São Paulo, SP, Brazil; d Health Technology Assessment Center, Cochrane Brazil, São Paulo, SP, Brazil; e Iberoamerican Cochrane Centre, Barcelona, Spain

**Keywords:** Clinical decision-making, Evidence-based practice, Health technology assessment, Rapid review

## Abstract

**Objective:**

To provide an overview of the activities and impact of an Evidence-Based Care Program (EBCP) on professionals’ decision-making in a large hospital located in São Paulo, Brazil.

**Methods:**

Retrospective descriptive analysis of a hospital EBCP's database (covering a 40-month period), involving data from the studies conducted and an online feedback survey.

**Results:**

Since its inception, the EBCP performed 95 scientific technical reports, including 55 Rapid Systematic Reviews (RSR) and 40 Literature Reviews (LR). The authors identified 21 review topics, with the 3 most common being inpatients (*n* = 20), oncology (*n* = 11), and surgical procedure (*n* = 9). The 3 most common technologies assessed were medical devices (*n* = 28), drugs (*n* = 27), and scale/questionnaire (*n* = 16). Studies were mainly used to update policy or procedure (*n* = 59), support scientific research (*n* = 10), support clinical program development (*n* = 8), provide clinical guidance (*n* = 7), or as a communication tool (*n* = 5). Among the requestors who responded to the feedback survey (76/95), 97 % agreed or strongly agreed that they were satisfied with the review delivered, and 100 % indicated that they likely would request a review in the future and would indicate the EBCP to a coworker. Among the RSR responders (51/55), 94 % agreed or strongly agreed that the EBCP work informed their project or final decision, and 32 % reported that the result presented changed their perspective about the technology assessed.

**Conclusion:**

Employing evidence-based methodology, the program provided timely and pertinent evidence for local decision-making. Health professionals who utilized the EBPC expressed high satisfaction with the process and reported a positive shift in their decision-making.

## Highlights

• Evidence-Based Care Program improves decision-making in a large Brazilian hospital.
• 95 reviews conducted, including 55 rapid and 40 scoping reviews in 40 months.
• High satisfaction: 99 % of requestors reported a positive impact on decision-making.
• Most frequent topics: medical devices, drugs, and scale assessments.
• Rapid reviews were critical for updating policies and guiding clinical decisions.

## Background

The application of evidence-based practices to support decision-making in procedure coverage, resource allocation, and clinical guidelines has expanded globally since the decade of 1970. Driven by economic and technological pressures, this process has become an essential aspect of health system governance worldwide.^1^

Over time, there has been increasing recognition that evaluations of health technologies must consider the specific contexts of individual organizations. As a result, many healthcare facilities are fostering a culture where clinical decisions are grounded in the best available evidence. This shift is accompanied by dedicated efforts to integrate scientific knowledge into everyday practice, motivating local hospitals to implement evidence-based decision-making processes.^1^^,^^2^

In the literature, various terms describe these initiatives, including the Evidence-Based Care (EBC) program, Evidence-Based Practice Center (EBPC), and Hospital-Based Health Technology Assessment (HB-HTA), among others. Collectively, these terms refer to a process designed to efficiently locate and synthesize research evidence, facilitating the integration of knowledge into institutional decision-making, and enhancing the quality, safety, and value of care provided.^1^^,^^3^, ^4^, ^5^

Despite the growing adoption of these practices, there remains a limited amount of evidence on the effectiveness and practical impact of such programs. Specifically, few studies have explored their utility from the perspective of stakeholders or their tangible influence on decision-making processes within healthcare organizations.^1^^,^^3^, ^4^, ^5^

This article introduces an EBC program implemented at BP – A Beneficência Portuguesa de São Paulo, a private nonprofit hospital organization in São Paulo, Brazil. The aim is to describe the program and present the activities conducted over a 40-month period.

## Methods

### Study design

This study presents a narrative report of the EBC program, along with a descriptive analysis of data from an internally maintained database, which includes the activities performed and feedback from requestors. All analyses were conducted retrospectively, covering data from the program's inception in August 2020 to December 2023. The data were analyzed descriptively, with the results imported into Microsoft® Excel® 365 software (version 2406).

### Setting

BP is a private nonprofit association with charitable, social, and scientific aims, headquartered in São Paulo, Brazil. In the fiscal year 2023, BP comprised seven hospital buildings and eleven independent clinics. The setting includes 32 surgical theaters, three of which are equipped with surgical robots, and 721 beds, with 166 designated for the Intensive Care Unit (ICU). The entire structure supports 52 clinical specialties, providing services to both adults and children with private or health plan coverage. Annually, BP's conducts approximately 35,900 surgeries, 41,600 chemotherapy sessions, and six million exams, along with about 30,500 consultations per month in the emergency department.

One of the fundamental pillars of BP is education and research. In this field, technology, knowledge exchange, and discoveries are integrated into decision-making and professional development. The synergy between education and research drives innovation and excellence within the healthcare setting. This approach enhances procedural standards, modernizes the care model, and cultivates skilled professionals.

### Description of the evidence-based care program

The EBC program, known as Observatory, was launched in August 2020 as part of BP's education and research department. Its primary objective is to integrate the best available evidence into institutional decision-making to enhance the quality and safety of patient care and optimize the value of care provided across the health system.

It is an interprofessional program, idealized by a physician with both clinical and administrative responsibilities, and staffed by two scientific consultants specializing in evidence-based health. This structure has been consistently maintained and is funded by the budget of BP's Chief Medical Officer. All reviews are performed for projects originating internally.

The program was based on Monash Health, a comprehensive e-health service network providing primary and secondary care in the southeast of Melbourne, Australia, as well as tertiary and quaternary care in specialist areas across Victoria, Australia.^6^ In our model, knowledge is translated through two primary methodologies: rapid reviews and scoping reviews. The first one is conducted in accordance with Cochrane guidelines^7^ while the second adheres to the Joanna Briggs Institute (JBI) guidelines.^8^ The methodology adopted depends on the research question.

Formal requests are received through a digital form hosted on the REDCap® platform,^9^ which manages the Observatory's database. Any BP collaborator is eligible to submit a research request. Upon submission, the consultant group evaluates the feasibility of the request within two business days. If the request is deemed feasible, a meeting with the requestor is scheduled to better understand the request, structure the research question and prioritize patient-centered outcomes.

The minimum projected timeline for completing a review is 15 business days from the start of the research. This timeline may vary considerably depending on the complexity of the question and the amount of available evidence. Once the review is completed, a meeting is held to present the findings. Subsequently, the technical report is uploaded to the REDCap® platform, which then sends an automated feedback survey two weeks later.

### Database analysis – activities performed

The internally maintained database is hosted on the REDCap® electronic data capture tools.^9^ For the analysis of activities performed, the authors categorized the demands based on the following criteria: (i) Type of research – rapid review or scoping review; (ii) Characteristics of the requestor – number of demands and department of work; (iii) Characteristics of the report – whether it is an empty review, includes meta-analysis, economic evidence or guideline/consensus, as well as the certainty of evidence; (iv) Technology assessed – drug, medical device, medical/surgical procedure, the process of care, scale, epidemiology or genomic analyses; (v) Clinical specialty examined – inpatient, outpatient, inpatient and outpatient, or another specialty (e.g., oncology, obstetrics etc.); and (vi) Goal of the research – basis for scientific research, clinical guidance outside of a policy or procedure, communication tool, resource allocation decision, support for the development of a clinical program, or update policy or procedure.

All variables were established internally, incorporating input from the EBC program group, decision-makers, and concepts from the literature. A brief explanation of each category and the topic within is presented in the supplementary file.

### Database analysis – feedback survey

The feedback survey is a standard procedure in the EBC program used to evaluate how well our recommendations align with users' expectations, assess their impact on decision-making, and gather suggestions for improving reports to better meet stakeholders' needs. This process is automated through the REDCap® platform,^9^ which sends a web-based survey and a copy of the requested report to the requestors two weeks after a report is completed and submitted. Participation is voluntary and anonymous.

The present survey is based on previous studies assessing the value of different EBC programs.^4^^,^^5^^,^^10^^,^^11^ The questionnaire comprises 12 items, with responses presented on a Likert scale^12^ of either three or five points, where higher numbers indicate greater agreement. A 13th open-ended question is presented at the end of the survey, allowing participants to provide free-text feedback regarding their experiences and suggestions for the program. The full questionnaire is available in the supplementary file.

For this study, the authors systematically analyzed feedback data from all requestors who received an EBC report between August 2020 and December 2023. Feedback was collected for each individual report, with questions tailored to the specific content of the corresponding report. Therefore, if a requestor had requested two reviews, for example, they received two distinct feedback surveys. This approach ensures that feedback is specific and relevant to each report, allowing for a precise evaluation of each report's impact and utility.

## Results

### Activities performed

From August 2020 to December 2023, the Observatory completed 95 requests, including 55 rapid reviews and 40 scoping reviews. During this period, all requested research was accepted and completed with an average turnaround time of 20 business days. The turnaround time varied slightly by review type, with rapid reviews generally having the longest duration.

Reviews were primarily requested by the Department of Medical Practices (*n* = 26), followed by the Department of Quality, Safety, and Care Practices (*n* = 12) and the Department of Education and Research (*n* = 11). In total, 42 individuals formalized a request. Of these, the majority (*n* = 34) submitted more than one request, with two individuals requesting up to nine reviews each during the study period.

Review topics varied over the 40-month period. Medical devices (*n* = 28) and drugs (*n* = 27) were the most examined, followed by scales (*n* = 16), epidemiology analyses (*n* = 10), medical procedures (*n* = 6), processes of care (*n* = 6), and genomic analyses (*n* = 2). Table 1 provides details on the type of technology assessed in each review topic.

**Table 1:** Type of technology assessed by review topic. BP – A Beneficência Portuguesa de São Paulo, 2020–2023 (*n* = 95).

**Review topic (n)**	**Technology assessed (n)**
Medical device, equipment or supplies (*n* = 28)	Surgical device (*n* = 7), extracorporeal therapy (*n* = 5), non-surgical device (*n* = 4), imaging techniques (*n* = 3), bandage (*n* = 2), clinical monitoring (*n* = 2), diagnostic techniques (*n* = 1), laser therapy (*n* = 1), minimally invasive procedure (*n* = 1), technology and information (*n* = 1), and venous access (*n* = 1).
Drug (*n* = 27)	Anesthetics (*n* = 3), intra-articular hyaluronic acid (*n* = 3), anticoagulants (*n* = 2), antimuscarinics (*n* = 2), hematopoietic growth factors (*n* = 2), multimedicines (*n* = 2), antidiarrheals (*n* = 1), antifibrotic (*n* = 1), antivirals (*n* = 1), cannabinoids (*n* = 1), GnRH agonists (*n* = 1), immunosuppressants (*n* = 1), immunotherapies (*n* = 1), intra-articular orthobiologics (*n* = 1), JAK inhibitors (*n* = 1), monoclonal antibodies (*n* = 1), monoethanolamine oleates (*n* = 1), NSAIDs (*n* = 1), and VEGF inhibitors (*n* = 1).
Test, scale, or risk factor (*n* = 16)	Clinical assessment (*n* = 14), and education and health management (*n* = 2).
Descriptive epidemiology (*n* = 10)	Incidence/prevalence (*n* = 9), and information and technology services (*n* = 1).
Medical/ Surgical procedure (*n* = 6)	Minimally invasive surgery (*n* = 2), surgical containment (*n* = 2), surgical procedure (*n* = 1), and venous access in surgery (*n* = 1).
Process of care (*n* = 6)	Care practice (*n* = 3), patient's safety (*n* = 1), extracorporeal procedure (*n* = 1), and clinical assessment (*n* = 1).
Genomic Analyses (*n* = 2)	Prognostic biomarkers (*n* = 2).

“n” denotes the number of requests completed for each respective topic.

GnRH, Gonadotropin-Releasing Hormone; JAK, Janus Kinase; NSAIDs, Non-Steroidal Anti-Inflammatory Drugs; VEGF, Vascular Endothelial Growth Factor.

**Fig. 1:**
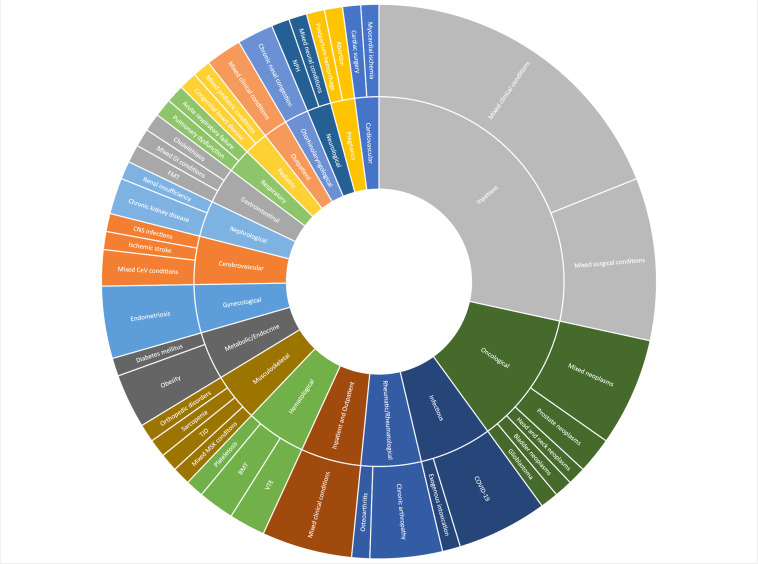
Distribution of requests by the type of health condition investigated. BP – A Beneficência Portuguesa de São Paulo, 2020–2023 (*n* = 95). Abbreviations: BMT, Bone Marrow Transplantation; CeV, Cerebrovascular; CNS, Central Nervous System; COVID-19, Coronavirus Disease 2019; FMT, Fecal Microbiota Transplantation; GI, Gastrointestinal; MSK, Musculoskeletal; NPH, Normal Pressure Hydrocephalus; TJD, Temporomandibular Joint Disorders; VTE, Venous Thromboembolism.

Fig. 1 shows the distribution of requests by the type of health condition investigated. Overall, inpatients (*n* = 27) and oncology (*n* = 11) were the most researched clinical specialties. Many specific conditions were subcategorized as “mixed conditions”. These included drugs, scales, medical devices, epidemiological analyses, medical procedures, and processes of care for multiple health conditions. For example, an antidiarrheal for magnetic resonance enterography or a questionnaire for clinical deterioration assessment.

Only a few requests resulted in an empty review (*n* = 5), and 22 included economic evidence. Meta-analysis was feasible in 37 reviews, and guidelines or consensus from international organizations were identified for 25 reports. The certainty of evidence was evaluated in 69 reviews, following the Grading of Recommendations Assessment, Development, and Evaluation (GRADE) system.^13^ Of these, the highest GRADE of evidence available for any comparison of interest was low or very low in most cases (*n* = 46), followed by moderate (*n* = 16) and high (*n* = 2).

The reviews were mainly requested to update an internal policy or procedure (*n* = 59). Other aims included: providing a basis for scientific research (*n* = 10), supporting the development of a clinical program (*n* = 8), offering clinical guidance outside of a policy or procedure (*n* = 7), assisting in resource allocation decisions (*n* = 6), and serving as a communication tool (*n* = 5).

### Feedback survey

Of the 95 requests performed over the 40-month period investigated, 76 requestors responded to the feedback survey, resulting in a response rate of 80 %. Among the respondents, 51 requested a rapid review and 25 requested a scoping review. Two data points were missing.

In general, respondents either agreed or strongly agreed that the reports were easy to request (*n* = 72), easy to understand (*n* = 76), addressed their doubts (*n* = 64), and were delivered within a timeframe that met their needs (*n* = 66). When asked about the report structure, most respondents indicated that the content was ideal (*n* = 60).

Significant positive responses were also obtained in the satisfaction section of the survey. Generally, respondents either agreed or strongly agreed that they were satisfied with the review delivered (*n* = 74), would recommend the Observatory to a colleague (*n* = 76), and were likely to request new reviews in the future (*n* = 76).

When respondents who requested a rapid review (*n* = 51) were asked about the influence of the request on their decision-making, most agreed or strongly agreed that the reports supported their final decision (*n* = 48) and that their final decision was consistent with the conclusions of the review (*n* = 47). Regarding the effect of the rapid review on their perspective before the request, most respondents indicated that it confirmed their initial perspective (*n* = 32).

Through a content analysis of the comments in the final section of the survey (*n* = 24), where participants could provide open feedback, the authors identified that the major criticism directed at the Observatory was the time taken to complete the requests. A small number of respondents, specifically two, mentioned that the process takes too long. However, several also highlighted that, despite the lengthy process, the final reports are detailed and reliable (*n* = 19). Moreover, most respondents (*n* = 22) expressed compliments and high satisfaction with the review provided.

## Discussion

Over a 40-month period, the EBC program completed nearly 100 reviews, encompassing both rapid and scoping methodologies. This substantial volume of reviews highlights robust engagement with the program, underscoring its perceived value within the institution. Notably, our program's average annual output compares favorably with similar studies, which reported annual averages of 30 to 64 reviews.^4^^,^^5^^,^^10^ Furthermore, the involvement of 42 individuals, many of whom submitted multiple requests, reflects the strong reliance on the Observatory's services for continuous improvement and informed decision-making.

A key strength of this program is its adherence to rigorous methodologies, such as those from the Cochrane group and the JBI. Unlike other EBC programs that sometimes deviate from strict methodologies,^4^^,^^5^^,^^10^ the unwavering commitment to these standards guarantees the reliability and credibility of our reviews. This adherence is made possible by our consultant staff, who are thoroughly trained in evidence synthesis and have substantial expertise in evidence-based health.

The diversity of review topics, ranging from medical devices and drugs to scales and epidemiology analyses, underscores the program's flexibility and wide-reaching impact across various clinical specialties. The majority of reviews focused on inpatients and oncology, which is not surprising given that BP is an advanced healthcare institution in Latin America, particularly recognized for its oncology services. This variety demonstrates the Observatory's capability to address complex and interdisciplinary questions, providing comprehensive reviews that support multiple aspects of patient-centered care and clinical practice.

The relative scarcity of high-quality evidence in the reports where GRADE analyses were conducted was expected, as it is documented that requestors are more likely to seek guidance when the evidence based on a topic is lacking.^4^^,^^5^^,^^10^ This was further supported by the small percentage of reports where sufficient homogeneous data existed to perform meta-analyses. The limited number of original meta-analyses conducted also reflects our reliance on secondary resources when available. These findings underscore the ongoing challenges in obtaining high-quality evidence for many healthcare interventions and highlight the importance of continuous research and methodological advancements to improve the certainty of available evidence.

Additionally, the low rate of economic analyses in the reviews highlights a significant challenge in incorporating cost considerations. Assessing costs can be difficult when published cost analyses are unavailable or do not reflect the hospital's perspective. This limitation underscores the need for more economic evaluations tailored to specific healthcare settings, providing a comprehensive understanding of the value of different interventions. However, in this experience, the few cost analyses that have been conducted, particularly in scenarios where robust evidence on health technologies was lacking ‒ played a critical role in decision-making and likely contributed to cost savings. However, these potential savings have not yet been documented in the institution.

The feedback survey results are particularly telling. Overall, they demonstrate that respondents were mainly satisfied with the reports, indicating that the reviews performed had a considerable impact on their decision-making processes. This feedback underscores the effectiveness of the Observatory in meeting the diverse needs of its users and affirms its role as a critical resource for informed decision-making within the institution.

Despite the overwhelmingly positive feedback, some respondents noted that the turnaround time for completing requests occasionally seemed lengthy. Notably, this is one of the primary challenges of EBC programs, where balancing adherence to rigorous evidence synthesis methods with the need for timely information delivery is crucial.^4^^,^^5^^,^^10^ To address this, the authors are currently exploring strategies to further streamline the review process. These might include expanding the team, leveraging technology for more efficient data synthesis, or prioritizing requests based on urgency and impact.

This retrospective analysis has some limitations. Firstly, the authors were unable to track whether the findings from the EBC reports translated into appropriate recommendations, whether these were implemented and adhered to, and ultimately, whether they led to improved patient outcomes. Although EBC reviews are vital for decision-making, their impact is influenced by factors such as economic considerations, implementation challenges across multiple facilities, and the need for innovation or competitive advantage.^4^ In the present study, the survey was totally dependent on the accuracy of the responses, which may introduce bias or inaccuracies in the reported data.

Furthermore, due to the focus of this study on the local evidence synthesis activities of our center, the authors did not include descriptions of our internal activities, such as multidisciplinary workshops for health professionals, article publications, and the preparation of internal documents, including formal demand forms and activity reports. Considering all these activities provides a more comprehensive understanding of the potential of EBC programs.

The study team also acknowledges a few limitations of the EBC program's process. One of these is the current dissemination of information from the reviews, which is mostly restricted to the requesting collaborator. To address this, the authors aim to ensure broader dissemination to maximize the impact of our reviews, both internally and publicly. Furthermore, the authors recognize that other programs have noted potential inefficiencies and waste associated with local centers producing duplicative reviews. A potential solution to this issue could be public sharing or establishing a central repository of rapid reviews. However, it remains uncertain whether reviews tailored to the local setting would be suitable for widespread sharing.^4^^,^^5^^,^^10^

To our knowledge, this is the first comprehensive description and assessment of evidence synthesis activity by a hospital EBC program in Brazil. Only a few hospitals and healthcare institutions in the United States of America have reported on their evidence synthesis activities, highlighting the importance of sharing experiences and methodologies from similar programs.^4^^,^^5^^,^^10^ Such studies can assist healthcare systems in identifying internal decisions that could benefit from locally sourced rapid reviews and in evaluating whether an in-house EBC program could enhance the value of care delivered.

## Conclusion

The findings from this study demonstrate that an EBC program using reputable review methodologies can successfully provide evidence-based syntheses to inform quality-of-care decision-making within a healthcare system. The program's value is highlighted by the substantial volume of reviews completed annually and the high satisfaction levels reported by requestors. Moreover, the program's sustainability and the consistent demand for reviews over a 40-month period underscore its critical role and utility within the organization. These results affirm the program's significance in enhancing healthcare delivery and supporting its continued development and integration into institutional practices.

## Authors' contributions

Conceptualization: ACPNP, CRRF, FSAR; Data curation: ACPNP, CRRF; Formal analysis: ACPNP, AR, CRRF; Investigation: ACPNP, AR, CRRF; Methodology: ACPNP, CRRF; Project administration: ACPNP, CRRF; FSAR, RMC; Supervision: FSAR, RMC; Validation: ACPNP, CRRF; Writing-original draft: ACPNP, AR, CRRF, FSAR, RMC; and Writing-review & editing: ACPNP, AR, CRRF, FSAR, RMC.

## Funding

This research did not receive any specific grant from funding agencies in the public, commercial, or not-for-profit sectors.

## Conflicts of interest

The authors declare no conflicts of interest.

## Appendix Supplementary materials

Image, application 1
