# Supplementary material for: An evidence-based care program in a local healthcare setting in Brazil: Experience and impact
Source: Clinics (Sao Paulo). 2025 Apr 16;80:100640. doi: 10.1016/j.clinsp.2025.100640 (PMC12284821; doi:10.1016/j.clinsp.2025.100640)
Supplement: Supplementary file 1 [file mmc1.docx]

**CLINICS-D-24-01066**

**SUPPLEMENTARY FILES**

**1** Variables established to categorize the demands performed.

| **Variable** | **Definition** |
| --- | --- |
| *Research type* | |
| Rapid review | As established by the Cochrane Methods Rapid Reviews Group.[1] |
| Scoping review | As established by the Joanna Briggs Institute (JBI) Scoping Review Methodology Group.[2] |
| *Requestor characteristics* | |
| Requested demands | A presentation of the total number of requests made by each collaborator throughout the study period. |
| Requester’s department | A presentation of the total number of requests made by each internal department within the clinical settings during the study period. |
| *Report characteristics* | |
| Empty review | A report indicated that no studies meeting the inclusion criteria were found.[3] |
| Meta-analysis | A report including a statistical combination of results from two or more separate studies.[4] |
| Economic evidence | A report including economic analyses or information on resource utilization and total costs.[5] |
| Guideline/consensus | A report incorporating established international guidelines or consensus statements. |
| Certainty of evidence | The degree of confidence in how closely an estimate of effect or association approximates the specific quantity of interest.[6] |
| *Technology categories* | |
| Descriptive epidemiology | A report primarily focused on evaluating the prevalence or incidence of clinical conditions, or the utilization of information technology. |
| Drug | A report primarily evaluating the benefits, safety, appropriate usage, or cost of a pharmacological agent.[7] |
| Genomic Analyses | A report primarily evaluating the genetic profile of specific health conditions, with a focus on identifying prognostic biomarkers and other genetic factors that may impact the diagnosis, treatment, and prognosis of diseases. |
| Medical device, equipment or supplies | A report primarily evaluating the benefits, safety, appropriate use, or cost of an instrument, apparatus, implement, machine, contrivance, implant, or related article intended for use in the prevention, diagnosis, or treatment of clinical conditions, excluding those achieving their primary purposes through chemical action or metabolism.[7] |
| Medical/surgical procedure | A report primarily evaluating the benefit, safety, appropriate use, or cost of a medical intervention that is neither a drug nor a device or evaluating the application or removal of a device.[7] |
| Process of care | A report primarily evaluating a clinical pathway or clinical practice guideline that significantly involves elements of prevention, diagnosis, and/or treatment, or that significantly incorporates two or more of the other technology categories.[7] |
| Test, scale, or risk factor | A report primarily evaluating the benefits, safety, appropriate use, or cost of a test designed to screen for, diagnose, classify, or monitor the progression of a disease or educational process.[7] |
| *Clinical specialty* | |
| Inpatient | A report primarily evaluating the management, treatment, and outcomes of patients in various hospital settings, including wards and Intensive Care Units (ICU). It may encompass multiple health conditions. |
| Outpatient | A report primarily evaluating the management, treatment, and outcomes of patients who are not admitted to the hospital, including those receiving care at home and in outpatient clinics. It may encompass multiple health conditions. |
| Inpatient and outpatient | A report primarily evaluating the management, treatment, and outcomes of diseases in both hospitalized and non-hospitalized patients, including epidemiological analyses of various conditions and evaluations of medications for different health conditions. It may encompass multiple health conditions. |
| Other specialty | A report primarily evaluating the interventions, treatments, or technologies relevant to specific medical specialties, such as oncology, cardiology, obstetrics etc. |
| *Goal of the research* | |
| Basis for scientific research | A report performed to summarize and analyze current evidence related to a specific topic or area, providing a comprehensive overview of existing literature and informing future research directions. |
| Clinical guidance outside of a policy or procedure | A report performed to provide clinical guidance and recommendations to healthcare professionals without implementing formal policy or procedural changes. |
| Communication tool | A report performed to update stakeholders on recent scientific advancements and new publications in a specific field. |
| Resource allocation deci­sion | A report performed to inform decisions about the allocation of resources among competing interventions or practices. |
| Support the development of a clinical program | A report performed to support the implementation of a new standard practice within the clinical setting. |
| Update policy or procedure | A report performed to revise or update existing clinical setting policies or procedures based on current evidence. |

**2** Feedback survey.

| **Record ID:** | | | | | | | | | | | | | | | | |
| --- | --- | --- | --- | --- | --- | --- | --- | --- | --- | --- | --- | --- | --- | --- | --- | --- |
| **1 Considerations about the report**  Please rate how much you agree or disagree with each statement below. | | | | | | | | | | | | | | | | |
| 1.1 I found it easy to request a report from the Observatory. | Strongly disagree | | | | Disagree | | | | | Neither agree nor disagree | | Agree | | | | Strongly agree |
|  | ⬜ | | | | ⬜ | | | | | ⬜ | | ⬜ | | | | ⬜ |
|  | | | | | | | | | | | | | | | | |
| 1.2 The report was easy to understand. | Strongly disagree | | | | Disagree | | | | | Neither agree nor disagree | | Agree | | | | Strongly agree |
|  | ⬜ | | | | ⬜ | | | | | ⬜ | | ⬜ | | | | ⬜ |
|  | | | | | | | | | | | | | | | | |
| 1.3 The report answered my doubts. | Strongly disagree | | | | Disagree | | | | | Neither agree nor disagree | | Agree | | | | Strongly agree |
|  | ⬜ | | | | ⬜ | | | | | ⬜ | | ⬜ | | | | ⬜ |
|  | | | | | | | | | | | | | | | | |
| 1.4 The deadline was adequate to my needs. | Strongly disagree | | | | Disagree | | | | | Neither agree nor disagree | | Agree | | | | Strongly agree |
|  | ⬜ | | | | ⬜ | | | | | ⬜ | | ⬜ | | | | ⬜ |
|  | | | | | | | | | | | | | | | | |
| **2 Report structure**  Please select the most appropriate answer to the following question: | | | | | | | | | | | | | | | | |
| 2.1 Briefly, how would you describe the content of the report? | | | | | | Very restricted | | | | | Ideal | | Very wide | | | |
|  |  |  |  |  |  | ⬜ | | | | | ⬜ | | ⬜ | | | |
|  | | | | | | | | | | | | | | | | |
| 2.2 The report was identified as a rapid review? | | | | | | Yes | | | | | No | |  | | | |
|  |  |  |  |  |  | ⬜ | | | | | ⬜ | |  | | | |
|  | | | | | | | | | | | | | | | | |
| **If Yes for 2.2* → 3 Decision-making considerations**  Please think about the final decision you made after reading the report. Rate how much you agree or disagree with each statement below. | | | | | | | | | | | | | | | | |
| 3.1 The report supported my final decision. | | | Strongly disagree | Disagree | | | | | | Neither agree nor disagree | | Agree | | | Strongly agree | |
|  |  |  | ⬜ | ⬜ | | | | | | ⬜ | | ⬜ | | | ⬜ | |
|  | | | | | | | | | | | | | | | | |
| 3.2 My final decision taken agreed with the report conclusions. | | | Strongly disagree | Disagree | | | | | | Neither agree nor disagree | | Agree | | | Strongly agree | |
|  |  |  | ⬜ | ⬜ | | | | | | ⬜ | | ⬜ | | | ⬜ | |
|  | | | | | | | | | | | | | | | | |
| Please select the most appropriate answer to the following question: | | | | | | | | | | | | | | | | |
| 3.3 Which of the following best describes the effect of the report on your perspective of the intervention prior to requesting the demand? | | | | | | | ⬜ | It had no effect on my perspective. | | | | | | | | |
|  |  |  |  |  |  |  | ⬜ | It confirmed my perspective. | | | | | | | | |
|  |  |  |  |  |  |  | ⬜ | It changed my perspective. | | | | | | | | |
|  | | | | | | |  |  | | | | | | | | |
|  | | | | | | |  |  | | | | | | | | |
| **4 Satisfaction with the report**  Please rate how much you agree or disagree with each statement below. | | | | | | | | | | | | | | | | |
| 4.1 Overall, I was satisfied with the report. | | Strongly disagree | | Disagree | | | | | Neither agree nor disagree | | | Agree | | Strongly agree | | |
|  |  | ⬜ | | ⬜ | | | | | ⬜ | | | ⬜ | | ⬜ | | |
|  | | | | | | | | | | | | | | | | |
| 4.2 I would recommend the Observatory to my colleagues. | | Strongly disagree | | Disagree | | | | | Neither agree nor disagree | | | Agree | | Strongly agree | | |
|  |  | ⬜ | | ⬜ | | | | | ⬜ | | | ⬜ | | ⬜ | | |
|  | | | | | | | | | | | | | | | | |
| 4.3 I will probably request new reports. | | Strongly disagree | | Disagree | | | | | Neither agree nor disagree | | | Agree | | Strongly agree | | |
|  |  | ⬜ | | ⬜ | | | | | ⬜ | | | ⬜ | | ⬜ | | |
|  | | | | | | | | | | | | | | | | |
| **5 Final considerations**  If you feel comfortable, leave suggestions, criticisms or observations here to improve our work. | | | | | | | | | | | | | | | | |
| *If no for 2.2 the platform goes directly to item 4. | | | | | | | | | | | | | | | | |

**References**

1. Garritty C, Hamel C, Trivella M, Gartlehner G, Nussbaumer-Streit B, Devane D, et al.; Cochrane Rapid Reviews Methods Group. Updated recommendations for the Cochrane rapid review methods guidance for rapid reviews of effectiveness. BMJ. 2024;384:e076335.

2. Peters MDJ, Marnie C, Tricco AC, Pollock D, Munn Z, Alexander L, et al. Updated methodological guidance for the conduct of scoping reviews. JBI Evid Synth. 2020;18(10):2119-26.

3. Yaffe J, Montgomery P, Hopewell S, Shepard LD. Empty reviews: a description and consideration of Cochrane systematic reviews with no included studies. PLoS One. 2012;7(5):e36626.

4. Deeks JJ, Higgins JPT, Altman DG (editors). Chapter 10: Analyzing data and undertaking meta-analyses. In: Higgins JPT, Thomas J, Chandler J, Cumpston M, Li T, Page MJ, Welch VA (editors). Cochrane Handbook for Systematic Reviews of Interventions version 6.4 (updated August 2023). Cochrane, 2023.

5. Aluko P, Graybill E, Craig D, Henderson C, Drummond M, Wilson ECF, et al.; on behalf of the Campbell and Cochrane Economics Methods Group. Chapter 20: Economic evidence. In: Higgins JPT, Thomas J, Chandler J, Cumpston M, Li T, Page MJ, Welch VA (editors). Cochrane Handbook for Systematic Reviews of Interventions version 6.4 (updated August 2023). Cochrane, 2023.

6. Schünemann H, Brożek J, Guyatt G, Oxman A, editors. GRADE handbook for grading quality of evidence and strength of recommendations. Updated October 2013. The GRADE Working Group, 2013.

7. Jayakumar KL, Lavenberg JA, Mitchell MD, Doshi JA, Leas B, Goldmann DR, et al.. Evidence synthesis activities of a hospital evidence-based practice center and impact on hospital decision making. J Hosp Med. 2016;11(3):185-92.
